# A Novel Anti-Cadherin-19 Monoclonal Antibody (Ca_19_Mab-8) for Flow Cytometry, Western Blotting, and Immunohistochemistry

**DOI:** 10.3390/cimb48030307

**Published:** 2026-03-12

**Authors:** Guanjie Li, Hiroyuki Suzuki, Mika K. Kaneko, Yukinari Kato

**Affiliations:** Department of Antibody Drug Development, Tohoku University Graduate School of Medicine, 2-1 Seiryo-machi, Aoba-ku, Sendai 980-8575, Miyagi, Japan; guanjie.li.e3@tohoku.ac.jp (G.L.); mika.kaneko.d4@tohoku.ac.jp (M.K.K.)

**Keywords:** Cadherin-19, CDH19, cell-based immunization and screening, monoclonal antibody, flow cytometry, immunohistochemistry

## Abstract

The type II cadherin Cadherin-19 (CDH19) plays a crucial role in neural crest development. CDH19 regulates cell–cell junctions and migration by forming catenin–cytoskeleton complexes. Although anti-CDH19 monoclonal antibodies (mAbs) are used for specific applications such as Western blotting and immunohistochemistry (IHC), suitable anti-CDH19 mAbs for flow cytometry are limited. Therefore, developing mAbs that specifically recognize cell-surface-expressed CDH19 is essential for advancing both basic research and therapeutic strategies. Here, novel anti-human CDH19 mAbs (Ca_19_Mabs) were created using flow cytometry-based high-throughput screening. One clone, Ca_19_Mab-8 (IgG_1_, κ), specifically recognized CDH19-overexpressed Chinese hamster ovary-K1 cells but did not bind to other 21 CDHs (including both type I and type II CDHs) in flow cytometry. Additionally, Ca_19_Mab-8 recognized endogenous CDH19 in the human glioblastoma cell line LN229. The dissociation constant (*K*_D_) of Ca_19_Mab-8 for LN229/CDH19 was 9.0 × 10^−9^ M. Ca_19_Mab-8 also detected endogenous CDH19 in Western blotting. Furthermore, Ca_19_Mab-8 can detect CDH19 in IHC using human melanoma tissue. These findings suggest that Ca_19_Mab-8 is a novel mAb that detects cell-surface-expressed CDH19 with high specificity and is suitable for various applications in basic research. Therefore, Ca_19_Mab-8 has potential for clinical diagnosis and tumor therapy.

## 1. Introduction

Cadherin-19 (CDH19) is a neural crest-specific adhesion molecule that plays a central role in maintaining brain homeostasis [1,2]. CDH19 belongs to the classical type II cadherin family and comprises five extracellular cadherin repeats (EC1–EC5), a single-pass transmembrane region, and a cytoplasmic tail [3,4,5]. The extracellular domain of CDH19 mediates calcium-dependent homophilic interactions at adherens junctions [6,7]. Its cytoplasmic domain associates with α-catenin, β-catenin, and p120-catenin, which link CDH19 to the actin cytoskeleton [8,9].

The human CDH19 gene was cloned in 2000 based on its sequence similarity to CDH7 [10]. Expressed sequence tags for CDH19 were isolated from melanocyte cDNA libraries, suggesting that CDH19 expression may be restricted to neural crest-derived cells [10]. Supporting this observation, rat CDH19 was primarily expressed in nerve ganglia and Schwann cells during embryonic development. Moreover, CDH19 expression overlapped with neural crest markers such as AP-2 and Sox10 [2]. In Sox10-knockout mouse embryos, neural crest cells exhibited delayed migration to the distal hindgut along with a simultaneous downregulation of CDH19 [1]. Furthermore, Sox10 was found to bind to the CDH19 promoter, indicating that CDH19 is a direct target of Sox10 during early neural crest cell migration by forming CDH19–catenin–cytoskeleton complexes [1]. Based on these functions, CDH19 is crucial for the development of neural crest cells, providing insights into the pathogenesis of nervous system developmental defects [11,12].

Melanoma is a type of skin cancer caused by the oncogenic transformation of melanocytes, which are pigment-producing skin cells derived from the neural crest [13,14]. Estimated new cases of melanoma in 2025 are more than 100,000 in the US [15], and approximately 20% of patients have advanced disease [American Joint Committee on Cancer (AJCC) stages: IIIA~IIID] at the time of diagnosis [16]. Although primary melanoma can be cured by surgery and immune checkpoint inhibitors are the standard of care for patients with advanced-stage melanoma [17,18], melanoma is the leading cause of death from skin disease in the US, responsible for 8430 estimated deaths in 2025 [15]. A single-cell multi-omics analysis of human melanoma revealed that melanoma cells were grouped into seven subtypes, which include a CDH19-high subtype, which was suggested to be more resistant to NK- and T-cell-mediated immunity [19].

Several monoclonal antibodies (mAbs) that detect CDH19 through Western blotting or immunohistochemistry (IHC) have been developed for various uses. Some of these recognize the intracellular domain of CDH19. Anti-CDH19 mAbs that target the extracellular domain and are available for flow cytometry are especially important for developing therapeutic mAbs. However, there have been no anti-CDH19 mAbs available for flow cytometry. Using the Cell-Based Immunization and Screening (CBIS) method, anti-E-Cadherin/CDH1 [20] and anti-M-Cadherin/CDH15 [21] mAbs for flow cytometry, Western blotting, and IHC were developed by our laboratory. The CBIS method includes high-throughput flow cytometry-based screening. Therefore, mAbs obtained by the CBIS method generally recognize conformational epitopes, enabling their use in flow cytometry. Some of the mAbs are suitable for Western blotting and IHC. In this study, we employed the CBIS method to develop highly versatile anti-CDH19 mAbs.

## 2. Materials and Methods

### 2.1. Cell Lines

Chinese hamster ovary (CHO)-K1, mouse myeloma P3X63Ag8U.1 (P3U1), and human glioblastoma (GBM) LN229 cells were obtained from the American Type Culture Collection (ATCC, Manassas, VA, USA).

### 2.2. Plasmid Construction and Establishment of Stable Transfectants

Genes encoding human *CDH19* (NM_021153) were purchased from OriGene Technologies, Inc. (Rockville, MD, USA). The *CDH19* cDNA without the pro-peptide was subcloned into the pCAG-Ble vector with an N-terminal PA16 tag [22]. Additionally, the *CDH19* cDNA with an N-terminal MAP16 tag [23] was constructed. These plasmids were transfected into CHO-K1 or LN229 cells via the Neon transfection system (Thermo Fisher Scientific, Inc., Waltham, MA, USA). The stable transfectants were sorted using the anti-PA16 tag mAb (clone NZ-1) [22], the anti-MAP16 tag mAb (clone PMab-1) [23], and a cell sorter (SH800, Sony Corp., Tokyo, Japan). Finally, PA16-CDH19-overexpressed CHO-K1 (CHO/CDH19) and MAP16-CDH19-overexpressed LN229 (LN229/CDH19) transfectants were established.

We previously established other CDH-overexpressed stable transfectants [24]. To confirm the expression of CDHs in these transfectants, 1 μg/mL of an anti-CDH1 mAb (clone 67A4), 1 μg/mL of an anti-CDH3 mAb (clone MM0508-9V11), or 0.1 μg/mL of an anti-PA16 tag mAb, NZ-33 [24] was used.

### 2.3. Production of Hybridomas

Female BALB/cAJcl mice (CLEA Japan, Tokyo, Japan) were intraperitoneally immunized with LN229/CDH19 cells (1 × 10^8^ cells/injection) mixed with a 2% Alhydrogel adjuvant (InvivoGen, San Diego, CA, USA). Following three additional weekly immunizations (1.0 × 10^8^ cells/injection), a booster dose (1 × 10^8^ cells/injection) was administered two days before spleen excision. Hybridomas were produced as previously described [21].

### 2.4. Flow Cytometry

A total of 1 × 10^5^ cells harvested with 1 mM EDTA were washed with phosphate-buffered saline (PBS) containing 0.1% bovine serum albumin (BSA; blocking buffer) and incubated with mAbs for 30 min at 4 °C. Flow cytometric data were collected on an SA3800 Cell Analyzer (Sony Corp.) by acquiring 5000 events. Cells were gated based on forward scatter (FSC) and side scatter (SSC), and fluorescence intensity was analyzed using FlowJo software (version 10.9.0, BD Biosciences, Franklin Lakes, NJ, USA).

### 2.5. Determination of Dissociation Constant Values Using Flow Cytometry

LN229/CDH19 cells were treated with serially diluted Ca_19_Mab-8, after which the saturating amount of Alexa Fluor 488-conjugated anti-mouse IgG (diluted 1:200) was added. The geometric mean (GeoMean) was calculated using FlowJo software. The binding isotherms [vertical axis, GeoMean (–background); horizontal axis, mAb concentration] were fitted to built-in one-site binding models in GraphPad Prism 6 (GraphPad Software, Inc., La Jolla, CA, USA) to determine the dissociation constant (*K*_D_) value.

### 2.6. Western Blotting

Western blotting was conducted using 1 μg/mL Ca_19_Mab-8 or a 1 μg/mL anti-isocitrate dehydrogenase 1 (IDH1) mAb (clone RcMab-1) as described previously [21].

### 2.7. IHC Using Cell Blocks and Tissue Arrays

All procedures of IHC were performed using VENTANA BenchMark ULTRA PLUS (Roche Diagnostics, Indianapolis, IN, USA). The formalin-fixed paraffin-embedded (FFPE) cell sections were prepared as described previously [21]. They are stained with 0.2 μg/mL Ca_19_Mab-8 or 0.01 μg/mL NZ-33 using the BenchMark ULTRA PLUS system along with the ultraView Universal DAB Detection Kit (Roche Diagnostics). Malignant melanoma (ME241a and ME242b) and glioblastoma (GL806e) tissue arrays (US Biomax Inc., Rockville, MD, USA) were stained with 2 μg/mL Ca_19_Mab-8 or 2 μg/mL isotype control IgG_1_ (CvMab-62).

## 3. Results

### 3.1. Anti-CDH19 mAb Development by the CBIS Method

**Figure 1:**
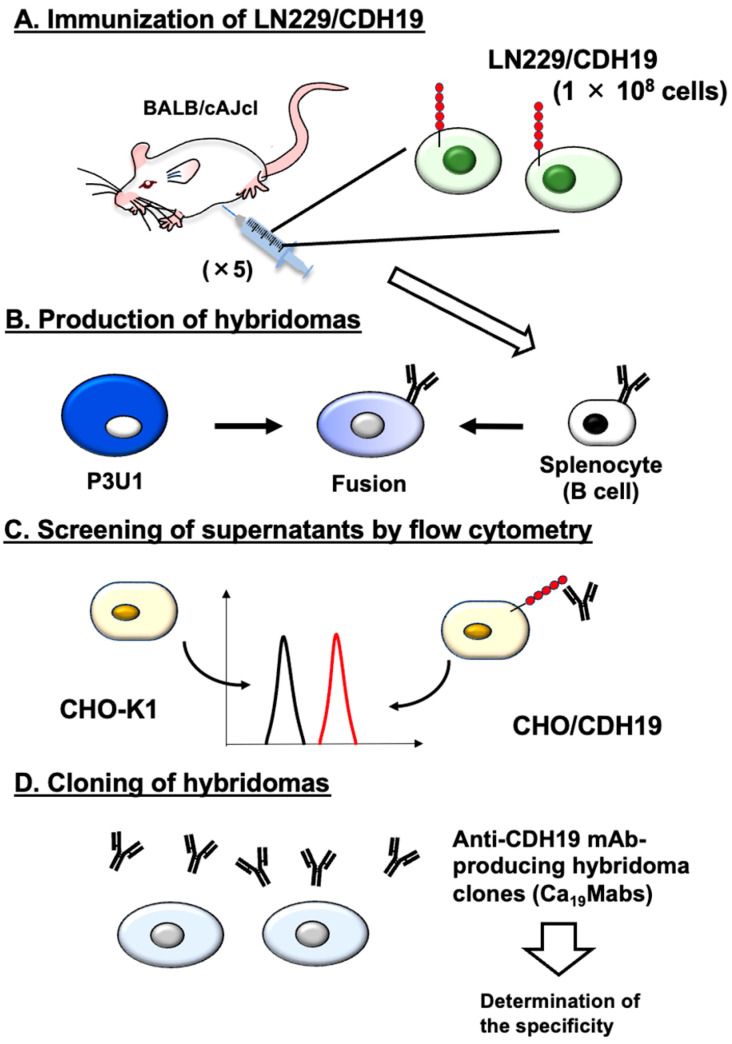
Schematic depiction of anti-CDH19 mAbs production. (**A**) LN229/CDH19 was injected into BALB/cAJcl mice intraperitoneally. (**B**) After five immunizations, spleen cells were fused with P3U1. (**C**) The supernatants from hybridomas were screened by flow cytometry using CHO/CDH19 and CHO-K1 cells. (**D**) Anti-CDH19 mAb-producing hybridoma clones (Ca_19_Mabs) were established through limiting dilution.

As described in the Materials and Methods Section, an immunogen, LN229/CDH19, was prepared. LN229/CDH19 (1 × 10^8^ cells/mouse) was immunized five times into two BALB/cAJcl mice (Figure 1A). Subsequently, hybridomas were generated by fusing splenocytes with myeloma P3U1 (Figure 1B). The hybridoma supernatants were screened to identify those positive for CHO/CDH19 and negative for CHO-K1 (Figure 1C). As a result, 43 positive wells out of 958 (4.5%) were found. Limiting dilution was then performed to clone hybridomas producing anti-CDH19 mAbs (Figure 1D). Ultimately, 12 clones were established (http://www.med-tohoku-antibody.com/topics/001_paper_antibody_PDIS.htm#CDH19, accessed on 9 March 2026), and the purified mAbs (IgG_1_ isotype) were prepared.

### 3.2. Determination of the Specificity of Ca_19_Mab-8 Using CDH-Overexpressed CHO-K1 Cells

**Figure 2:**
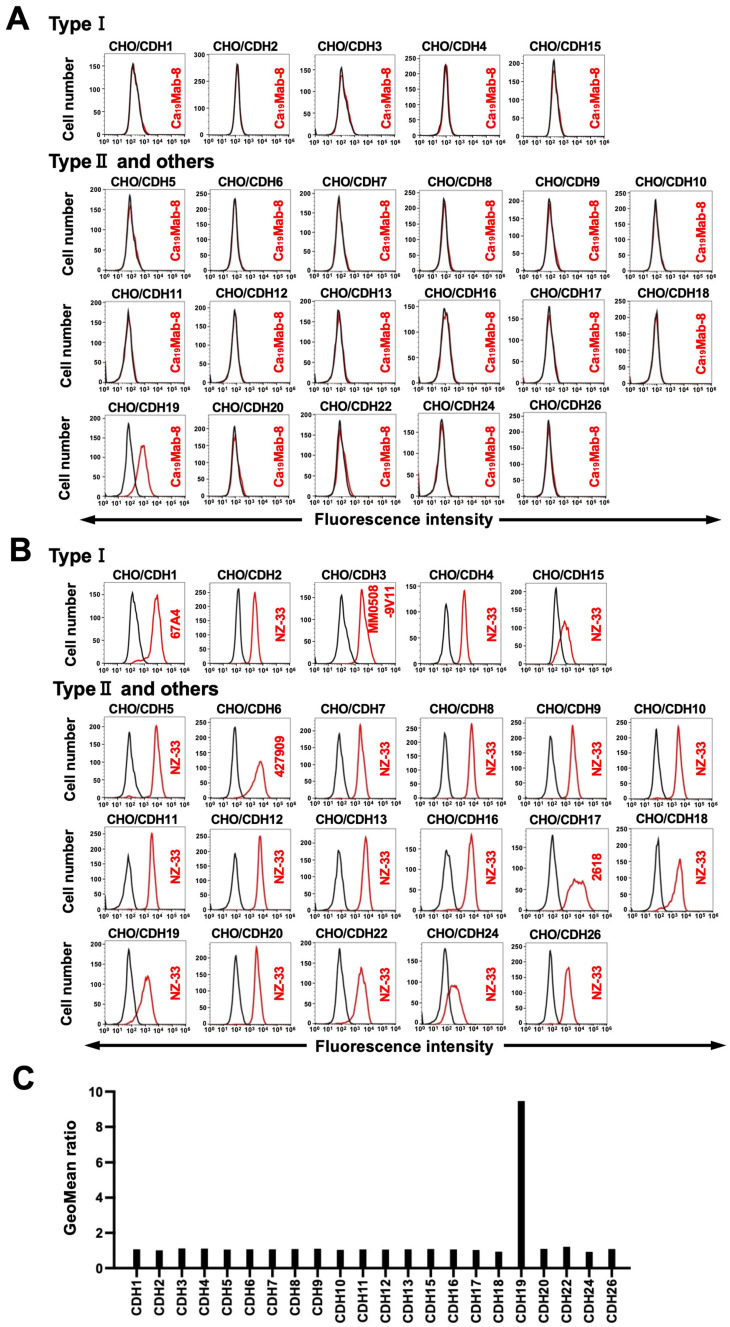
Flow cytometry analysis of Ca_19_Mab-8 in CDH-overexpressed CHO-K1 cells. (**A**) The type I CDHs (CDH1, CDH2, CDH3, CDH4, and CDH15), type II CDHs (CDH5, CDH6, CDH7, CDH8, CDH9, CDH10, CDH11, CDH12, CDH18, CDH19, CDH20, CDH22, and CDH24), 7D CDHs (CDH16 and CDH17), a truncated CDH (CDH13), and an atypical CDH (CDH26)-overexpressed CHO-K1 cell were treated with 10 µg/mL Ca_19_Mab-8 (red) or with a control blocking buffer (black, negative control), followed by treatment with anti-mouse IgG conjugated with Alexa Fluor 488. (**B**) Each CDH expression was confirmed by a 1 µg/mL anti-CDH1 mAb (clone 67A4, BD Biosciences), a 1 µg/mL anti-CDH3 mAb (clone MM0508-9V11, Abcam, Waltham, MA, USA), and a 1 µg/mL anti-PA16-tag mAb (clone NZ-33) to detect other CDHs, followed by treatment with Alexa Fluor 488-conjugated secondary mAbs. The fluorescence data were collected using the SA3800 Cell Analyzer. The experiments were conducted at least twice. The representative graphs are shown. (**C**) The GeoMean ratio relative to the negative control in (**A**) was determined.

We previously established CHO-K1 cells that overexpressed type I CDHs (CDH1, CDH2, CDH3, CDH4, and CDH15) [20,21], type II CDHs (CDH5, CDH6, CDH7, CDH8, CDH9, CDH10, CDH11, CDH12, CDH18, CDH19, CDH20, CDH22, and CDH24), 7D CDHs (CDH16 and CDH17), a truncated CDH (CDH13), and an atypical CDH (CDH26) [24]. Therefore, the specificity of Ca_19_Mabs to those CDHs was determined. As shown in Figure 2A, Ca_19_Mab-8 reacted with CHO/CDH19 but did not react with other CDHs overexpressed in CHO-K1 cells. The cell surface expression of CDHs was confirmed in Figure 2B. In contrast, other Ca_19_Mabs showed cross-reactivity against other type II CDHs (Table 1). These results indicate that Ca_19_Mab-8 is a specific mAb to CDH19 among those CDHs.

### 3.3. Flow Cytometry of Ca_19_Mab-8 Against CDH19-Overexpressed CHO-K1 and LN229 Cells

**Figure 3:**
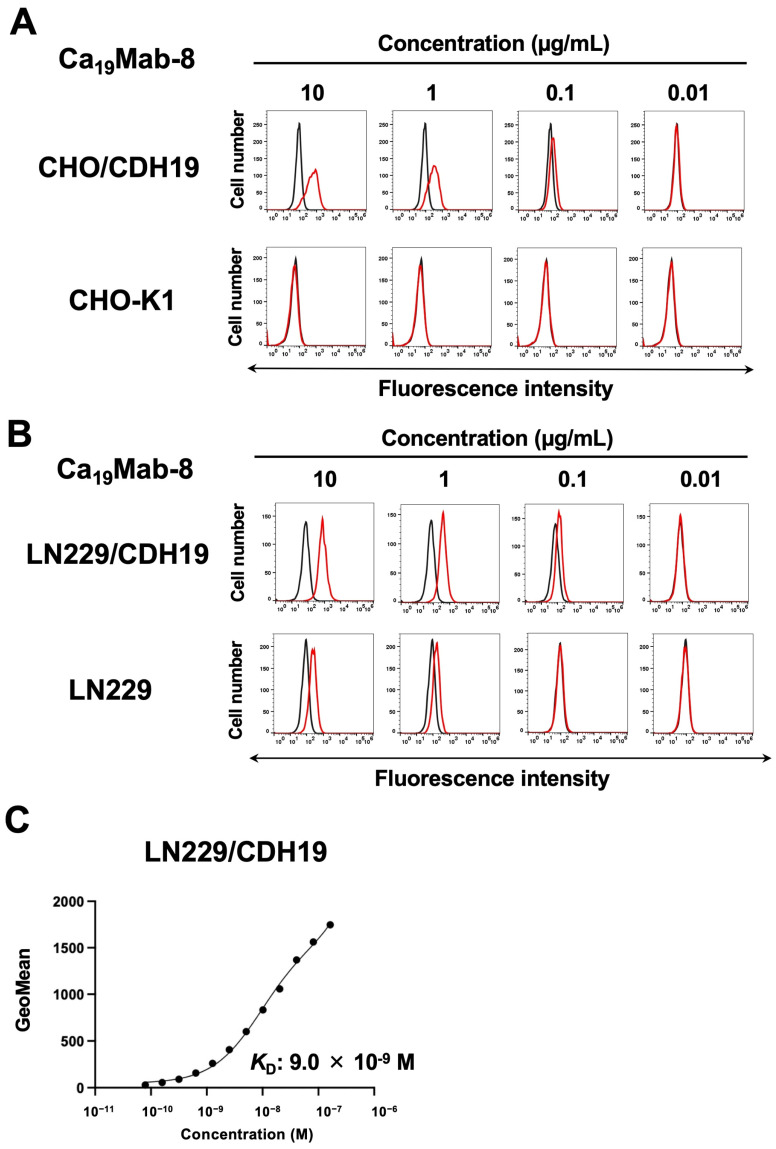
Flow cytometric analysis of Ca_19_Mab-8. (**A**) CHO/CDH19 and CHO-K1 were treated with Ca_19_Mab-8 at the indicated concentrations (red) or with a blocking buffer (black, negative control). (**B**) LN229/CDH19 and LN229 were treated with Ca_19_Mab-8 at the indicated concentrations (red) or with a blocking buffer (black, negative control). The mAb-treated cells were incubated with Alexa Fluor 488-conjugated anti-mouse IgG. Fluorescence data were collected using the SA3800 Cell Analyzer. (**C**) LN229/CDH19 was suspended in 100 µL of serially diluted Ca_19_Mab-8. The cells were treated with Alexa Fluor 488-conjugated anti-mouse IgG. Subsequently, the values of the geometric mean from fluorescence data were collected. The *K*_D_ value was calculated using GraphPad PRISM 6 software. The experiments were conducted at least twice. The representative images and graph are shown.

We next conducted flow cytometry using Ca_19_Mab-8 (IgG_1_, κ) against CHO/CDH19, CHO-K1, LN229/CDH19, and LN229. Ca_19_Mab-8 reacted with CHO/CDH19 in a dose-dependent manner, from 10 to 0.01 μg/mL (Figure 3A). In contrast, Ca_19_Mab-8 did not recognize CHO-K1 at 10 μg/mL (Figure 3A). Furthermore, Ca_19_Mab-8 reacted with LN229/CDH19 in a dose-dependent manner (Figure 3B). We also confirmed that the isotype control mAb (CvMab-62) did not recognize CHO/CDH19 and LN229/CDH19 (Supplementary Figure S1). Ca_19_Mab-8 also showed reactivity to parental LN229, suggesting that LN229 cells expressed endogenous CDH19. The binding affinity of Ca_19_Mab-8 was measured using flow cytometry. The fitting binding isotherms of Ca_19_Mab-8 to LN229/CDH19 are shown in Figure 3C. The *K*_D_ value of Ca_19_Mab-8 for LN229/CDH19 was 9.0 × 10^−9^ M. These results indicate that Ca_19_Mab-8 possesses a moderate binding affinity to LN229/CDH19.

### 3.4. Western Blotting Using Ca_19_Mab-8

**Figure 4:**
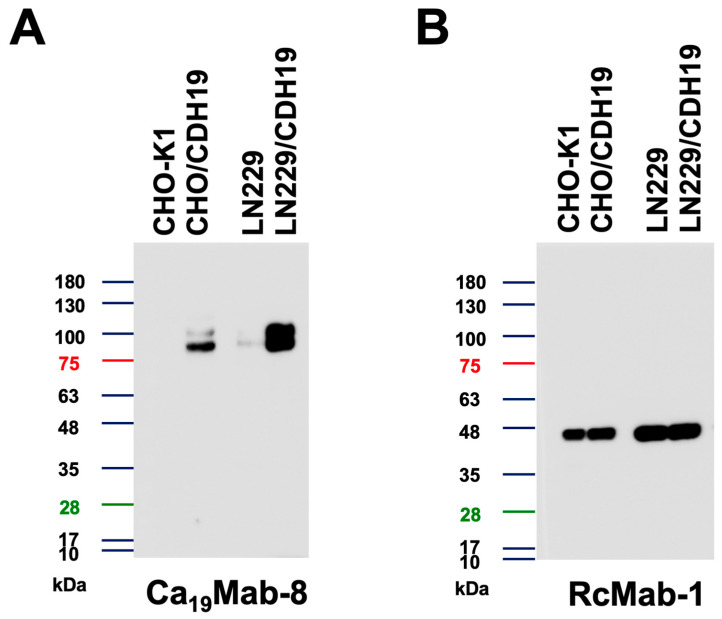
Western blotting using Ca_19_Mab-8. Cell lysates (10 μg/lane) from CHO-K1, CHO/CDH19, LN229, and LN229/CDH19 were electrophoresed and transferred to polyvinylidene difluoride membranes. The membranes were incubated with 1 μg/mL Ca_19_Mab-8 (**A**) or 1 μg/mL RcMab-1 (an anti-IDH1 mAb) (**B**), followed by treatment with anti-mouse (Ca_19_Mab-8) or anti-rat IgG (RcMab-1) conjugated with horseradish peroxidase. The experiments were conducted at least twice. The representative images are shown.

We then examined whether Ca_19_Mab-8 is suitable for Western blotting. Whole-cell lysates from CHO-K1, CHO/CDH19, LN229, and LN229/CDH19 were analyzed. Ca_19_Mab-8 detected clear bands around 90–100 kDa in CHO/CDH19 and LN229/CDH19 but not in CHO-K1 (Figure 4A). It also detected bands in parental LN229, approximately 1/20 of LN229/CDH19, as shown in Figure 4A. Figure 4B shows an internal control, IDH1, detected by RcMab-1. These results demonstrate that Ca_19_Mab-8 can detect both exogenous and endogenous CDH19 via Western blotting.

### 3.5. IHC Using Ca_19_Mab-8 in Formalin-Fixed Paraffin-Embedded Cell Blocks

**Figure 5:**
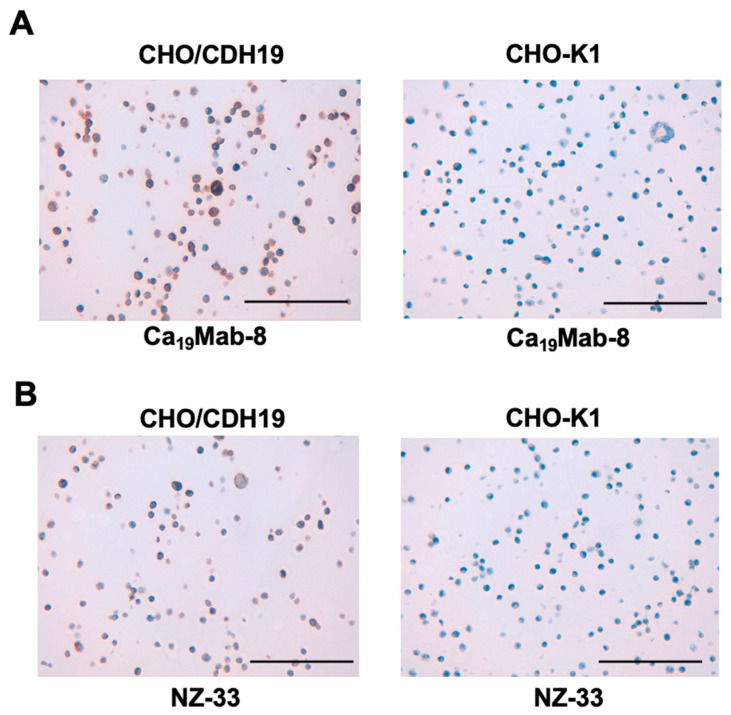
Immunohistochemistry using Ca_19_Mab-8 in formalin-fixed paraffin-embedded cell blocks. CHO/CDH19 and CHO-K1 sections were treated with 0.2 μg/mL Ca_19_Mab-8 (**A**) or 0.01 µg/mL NZ-33. (**B**) The staining was performed using BenchMark ULTRA PLUS with the ultraView Universal DAB Detection Kit (scale bar = 100 μm). The experiments were conducted at least twice. The representative images are shown.

We assessed whether Ca_19_Mab-8 is suitable for immunohistochemistry (IHC) of FFPE sections from CHO-K1 and CHO/CDH19 cells. Ca_19_Mab-8 showed strong cytoplasmic and membranous staining in CHO/CDH19 but not in CHO-K1 (Figure 5A). Additionally, an anti-PA16 tag mAb, NZ-33, exhibited cytoplasmic and membranous staining in CHO/CDH19 but not in CHO-K1 (Figure 5B). These results suggest that Ca_19_Mab-8 can detect CDH19 via IHC of FFPE sections of cultured cells.

### 3.6. IHC Using Ca_19_Mab-8 in Formalin-Fixed Paraffin-Embedded Tumor Tissue

**Figure 6:**
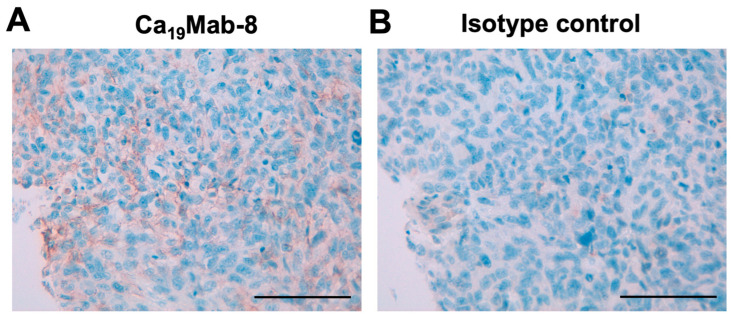
Immunohistochemistry using Ca_19_Mab-8 in the melanoma tissue array. Sequential sections of the human melanoma tissue array was treated with 2 μg/mL Ca_19_Mab-8 (**A**) or 2 μg/mL isotype control IgG_1_ (CvMab-62). (**B**) Staining was performed using BenchMark ULTRA PLUS with the ultraView Universal DAB Detection Kit (scale bar = 100 μm).

We next performed immunohistochemistry (IHC) of Ca_19_Mab-8 using glioblastoma (GL806e) and malignant melanoma (ME241a and ME242b) tissue arrays. Ca_19_Mab-8 showed membranous staining in one case of rectal malignant melanoma out of 10 cases in ME241a, while the isotype control mAb did not (Figure 6 and Table 2). Although another malignant melanoma tissue array (ME242b) was examined, we did not find CDH19-positive melanoma in any of the 12 cases. Additionally, no CDH19-positive cases were observed in the glioblastoma tissue array, which contained 40 cases. Representative images are shown in Supplementary Figure S2. These results suggest that Ca_19_Mab-8 is suitable for detecting endogenous CDH19 in FFPE tumor tissues by IHC.

## 4. Discussion

This study identified novel anti-CDH19 mAbs using the CBIS method (Figure 1). Among them, Ca_19_Mab-8 recognized both exogenous and endogenous CDH19 in flow cytometry and Western blotting (Figure 3 and Figure 4). Ca_19_Mab-8 specifically binds to CDH19 but not to other type II, type I, 7D, truncated, or atypical CDHs (Figure 2). In contrast, the other nine Ca_19_Mabs showed cross-reactivity with CDH7 and other type II CDHs in flow cytometry (Table 1). Since most commercially available mAbs lack information on cross-reactivity, careful handling and interpretation are essential when using them. Identifying the Ca_19_Mab-8 epitope is crucial for developing more specific anti-CDH19 mAbs. Additionally, Ca_19_Mab-8 is suitable for IHC on cell blocks (Figure 5) and tissue array (Figure 6). IHC was performed using an automated slide-staining system, allowing for standardized staining conditions for tumor diagnosis. Ca_19_Mab-8 is highly versatile for both basic research and clinical applications.

We demonstrated that Ca_19_Mab-8 recognized human GBM LN229 cells in flow cytometry and Western blotting (Figure 3 and Figure 4). Although we examined the reactivity of Ca_19_Mab-8 in other glioblastoma cell lines, LN229 is the only cell line identified by Ca_19_Mab-8. Since the expression of CDH19 in LN229 is not high, there is a limitation to its use for in vivo research. Therefore, it is essential to find a CDH19-overexpressing cancer cell line in the future research. CDH19 was detected in GBM stem-like cells isolated from fresh GBM samples [25]. We should investigate the reactivity of Ca_19_Mab-8 with those samples and assess its antitumor efficacy. We previously cloned the cDNA of mAbs and produced recombinant mouse IgG_2a_-type mAbs to confer antibody-dependent cellular cytotoxicity (ADCC). These mAbs have been evaluated for their antitumor efficacy in human tumor xenograft models [26,27]. We have cloned the cDNA of Ca_19_Mab-8, and recombinant Ca_19_Mab-8 will be produced and evaluated for its in vitro ADCC activity and antitumor activity in mouse glioblastoma xenograft models.

Metastatic melanoma remains a major clinical challenge [28,29]. Although therapeutic outcomes have significantly improved since the introduction of immune checkpoint inhibitors, about half of the patients with metastatic melanoma do not achieve long-term survival benefits [30,31]. A recent single-cell and spatial multi-omics analysis showed that drug-naive human melanoma biopsies contain cancer cells in a mesenchymal-like (MES) state [32]. Melanoma cells in the MES state exhibit resistance to targeted therapy and immunotherapy and are more frequently found in lesions that do not respond to treatment [32].

Ca_19_Mab-8 showed membranous staining of CDH19 in a case of rectal malignant melanoma (Figure 6). Recently, CDH19 was identified as an MES signature regulated by the master transcription factor TCF4. Targeting TCF4 genetically enhances immunogenicity and increases the sensitivity of MES cells to immunotherapy and targeted treatments [32]. Therefore, CDH19 is a promising antigen for targeting drug-resistant melanoma cells in the MES state. Moreover, a patent (US 2025/0084160 A1, 13 March 2025) reported that CDH19 is expressed in melanoma cell lines, such as CHL-1, which can serve as a preclinical model for melanoma therapy. Since Ca_19_Mab-8 can detect CDH19-positive cells by flow cytometry (Figure 3) and IHC (Figure 6) without cross-reactivity (Figure 2), Ca_19_Mab-8 could serve as a crucial foundation for developing various modalities, including antibody–drug conjugates, bispecific antibodies, and chimeric antigen receptor T cells. Additional validations such as mAb internalization and epitope accessibility are vital for therapeutic use.

## Figures and Tables

**Table 1:** Cross-reactivity of Ca_19_Mabs (IgG_1_ isotype) in flow cytometry.

Ca_19_Mabs	Isotype	Cross-Reacted CDHs in Flow Cytometry
Ca_19_Mab-1	IgG_1_, κ	CDH9
Ca_19_Mab-2	IgG_1_, κ	CDH6, CDH9, CDH 20
Ca_19_Mab-3	IgG_1_, κ	CDH6, CDH9
Ca_19_Mab-5	IgG_1_, κ	CDH7, CDH8, CDH11, CDH12, CDH18, CDH20, CDH22, CDH24
Ca_19_Mab-6	IgG_1_, κ	CDH7, CDH8, CDH9, CDH11, CDH12, CDH18, CDH20, CDH22 CDH24
Ca_19_Mab-7	IgG_1_, κ	CDH7, CDH8, CDH9, CDH10, CDH11, CDH12, CDH18, CDH22
Ca_19_Mab-8	IgG_1_, κ	− *^1^
Ca_19_Mab-9	IgG_1_, κ	− *^2^
Ca_19_Mab-11	IgG_1_, κ	CDH8, CDH9, CDH11, CDH12, CDH18, CDH22
Ca_19_Mab-12	IgG_1_, κ	CDH9, CDH20

Cross-reactivity was determined by flow cytometry using other type II CDHs (CDH5, CDH6, CDH7, CDH8, CDH9, CDH10, CDH11, CDH12, CDH18, CDH20, CDH22, and CDH24), 7D CDHs (CDH16 and CDH17), and a truncated CDH (CDH13)-overexpressed CHO-K1. *^1^ Ca_19_Mab-8 did not show the cross-reactivity (presented in Figure 2). *^2^ Ca_19_Mab-9 showed a reactivity to parental CHO-K1.

**Table 2:** Immunohistochemistry using Ca_19_Mab-8 against the melanoma tissue array (ME241a).

Age	Sex	Pathology Diagnosis	TNM	Ca_19_Mab-8
50	M	Malignant melanoma of the esophagus	-	-
62	F	Malignant melanoma of the right thumb	T4N0M0	-
70	F	Malignant melanoma of the parotid gland	T4N0M0	-
57	M	Malignant melanoma of the rectum	-	+
67	F	Malignant melanoma of the rectum	-	-
70	F	Malignant melanoma of the anus	T4N0M0	-
66	M	Malignant melanoma of the rectum	-	-
54	F	Malignant melanoma of the rectum	-	-
82	F	Malignant melanoma of the rectum	-	-
52	F	Malignant melanoma of the rectum	-	-
49	F	Cancer adjacent normal chest skin tissue	-	-
50	F	Cancer adjacent normal skin tissue	-	-

-, No staining; +, Positive staining.

## Data Availability

The original contributions presented in this study are included in the article/Supplementary Materials. Further inquiries can be directed to the corresponding authors.

## Supplementary Materials

The following supporting information can be downloaded at https://www.mdpi.com/article/10.3390/cimb48030307/s1.
