# Supplementary material for: A Novel Anti-Cadherin-19 Monoclonal Antibody (Ca19Mab-8) for Flow Cytometry, Western Blotting, and Immunohistochemistry
Source: Curr Issues Mol Biol. 2026 Mar 12;48(3):307. doi: 10.3390/cimb48030307 (PMC13026002; doi:10.3390/cimb48030307)
Supplement: Supplementary file 1 [file cimb-48-00307-s001.zip › cimb-4183612-supplementary.pdf]

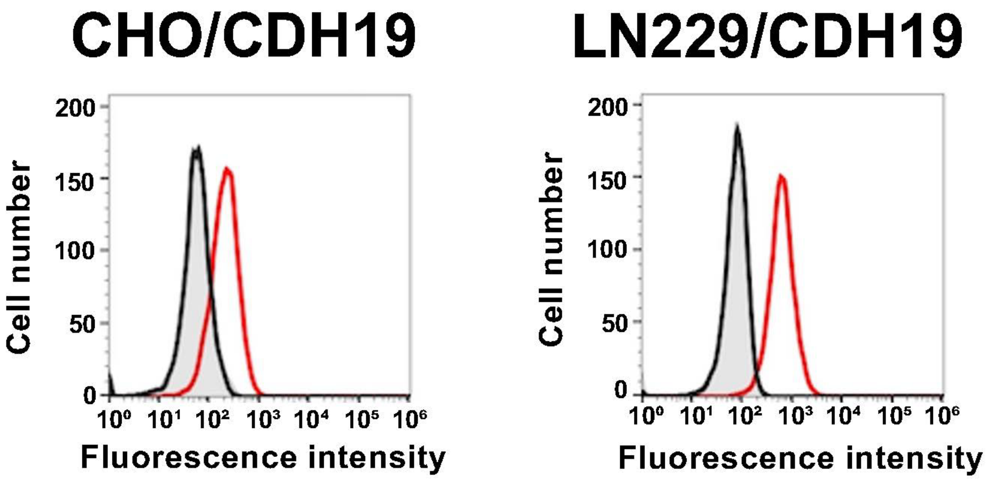

Supplementary Figure S1. Flow cytometric analysis using an isotype control mAb. CHO/CDH19 and LN229/CDH19 were treated with 1 μg/mL of CvMab-62, an isotype control IgG$_1$ mAb (filled gray), 1 μg/mL of Ca$_{19}$Mab-8 (red), or blocking buffer (black, negative control). The mAbs-treated cells were incubated with anti-mouse IgG conjugated with Alexa Fluor 488. The fluorescence data were collected using the SA3800 Cell Analyzer.

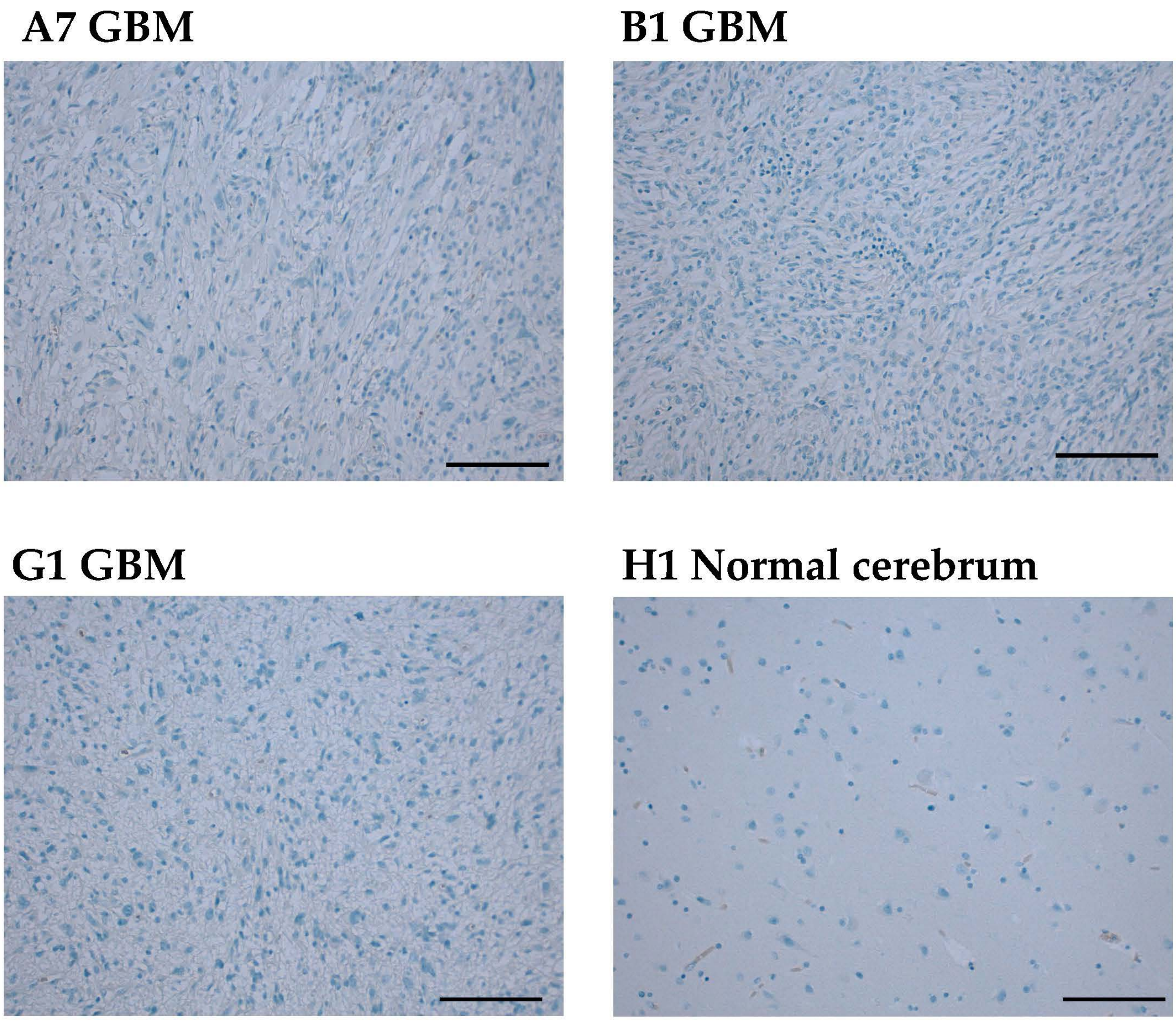

Supplementary Figure S2. Immunohistochemistry using Ca$_{19}$Mab-8 in formalin-fixed paraffin-embedded glioblastoma (GBM) tissue array. A human GBM tissue array was treated with 2 μg/mL of Ca$_{19}$Mab-8. The staining was performed using BenchMark ULTRA PLUS with the ultraView Universal DAB Detection Kit, Scale bar = 100 μm.
